# Descriptive Analysis of Pediatric Studies Included in the European Union Post-Authorization Study Register from 2010 to 2023

**DOI:** 10.3390/pediatric17010024

**Published:** 2025-02-16

**Authors:** Annalisa Landi, Giorgio Reggiardo, Antonella Didio, Annunziata D’Ercole, Adriana Ceci, Grace Shalom Govere, Donato Bonifazi, Fedele Bonifazi, Salvatore Crisafulli, Gianluca Trifirò, Florentia Kaguelidou, Katja Marja Hakkarainen, Katarina Gvozdanović, Francesco Barone-Adesi, Andrealuna Ucciero, Mariagrazia Felisi

**Affiliations:** 1 TEDDY European Network of Excellence for Pediatric Research, 27100 Pavia, Italy; giorgioreggiardo@cvbf.net (G.R.); adriceci.uni@gmail.com (A.C.); ceo@cvbf.net (D.B.); fb@benzifoundation.org (F.B.); mfelisi@cvbf.net (M.F.); 2 Fondazione per la Ricerca Farmacologica Gianni Benzi Onlus, 70124 Bari, Italy; antonella.didio92@gmail.com (A.D.); nde@benzifoundation.org (A.D.); 3 CVBF–Consorzio per Valutazioni Biologiche e Farmacologiche, 70122 Bari, Italy; grace-shalom.govere@unamur.be; 4 Department of Diagnostics and Public Health, University of Verona, 37134 Verona, Italy; salvatore.crisafulli@univr.it (S.C.); gianluca.trifiro@univr.it (G.T.); 5 Assistance Publique–Hôpitaux de Paris, Hôpital Universitaire Robert-Debré Centre d’Investigations Cliniques et d’Epidémiologie Clinique, 75019 Paris, France; florentia.kaguelidou@aphp.fr; 6 Parexel International, Epidemiology and Real-World Evidence, 41123 Gothenburg, Sweden; katja.hakkarainen@parexel.com; 7 Pharmacoepidemiology Department, Teaching Institute of Public Health “Dr. Andrija Stampar”, Zagreb 10 000, Croatia; katarina.gvozdanovic@stampar.hr; 8 Department of Translational Medicine, University of Eastern Piedmont, 28100 Novara, Italy; francesco.baroneadesi@uniupo.it; 9 Hospital Pharmacy, University Hospital Maggiore della Carità, 28100 Novara, Italy; andrealuna.ucciero@uniupo.it

**Keywords:** Post-Authorization Studies, pediatric, EU PAS Register, descriptive analysis

## Abstract

Background/Objectives: This work aimed to analyze pediatric Post-Authorization Studies (PASs) registered in the European Union electronic Register of Post-Authorization Studies (EU PAS Register) from September 2010 to April 2023 to identify trends in terms of timing, age groups, and therapeutic areas and to discuss pediatric specificities and sources of funding for the PASs. Methods: A screening process identified PASs conducted exclusively on the pediatric population, and instructions were provided to ensure standardized data collection from the EU PAS Register. A univariate linear regression descriptive analysis was performed to assess trends over time, while a multivariate linear regression analysis helped explore additional characteristics of these studies. Results: Of the 2574 PASs extracted from the EU PAS Registry, 165 were included in this analysis. The majority of pediatric PASs were observational studies (86%), and most of them utilized secondary data (53%). The annual number of PASs increased significantly between 2010 and 2023. As envisaged, the largest part was funded by pharmaceutical companies (62%). Anti-infectives for systemic uses (25%), medicines for the nervous system (18%), and antineoplastic and immunomodulating agents (15%) resulted in the most studied drugs. Conclusions: Our findings show that post-marketing observational research in pediatric populations has increased over time. Nevertheless, industry–academia collaboration should be encouraged, and regulatory guidance is needed to prioritize research in areas of unmet therapeutic need.

## 1. Introduction

The evaluation of drug efficacy and safety is a long-lasting process that continues after marketing authorization has been granted. In Europe, post-authorization requirements and commitments may include specific obligations/legally binding measures or recommendations or additional pharmacovigilance activities detailed in the risk management plan of the medicinal product, which are all aimed at obtaining additional effectiveness and safety data [1]. Moreover, other post-approval studies, including those conducted by industries and independent investigators, should be considered part of the ongoing, continuous evaluation efforts [2].

Post-Authorization Studies (PASs) play a crucial role in the post-authorization phase of a drug, confirming its benefit–risk profile in real-world settings and helping to detect long-term or rare adverse events that may not have been identified in pre-authorization clinical trials.

In the post-authorization phase, it may be necessary to collect additional data on the safety, especially the long-term effects, and, in certain cases, the “real-world efficacy”, effectiveness by investigating how well the treatment works in practice, or quality of authorized medicines to complement the available pre-marketing data [3]. Concerning safety, PASs include those to identify, characterize, or quantify a safety hazard and to confirm the safety profile of the authorized medicinal product or measure the effectiveness of risk management measures (Post-Authorization Safety Studies, PASS). Concerning efficacy, PASs include studies aimed at supplementing available efficacy data coming from pre-marketing clinical trials in the light of well-founded scientific uncertainties on aspects of the proof of benefit that should or can only be addressed after the medicinal product has been approved (Post-Authorization Efficacy Studies, PAES).

Although PASs primarily adopt a non-interventional approach (i.e., observational studies), they may also use interventional study designs (i.e., clinical trials) [4].

PASs are designed for different purposes from pre-marketing studies. Their designs are not systematically submitted to regulatory authorities prior to initiation because many PASs are conducted by independent investigators, and their conduct is less rigorously regulated. In addition, pre-marketing studies are almost exclusively sponsored by manufacturers, whereas PASs may be funded not only by manufacturers but also by academic or other types of not-for-profit institutions. Some research suggests that many PASs are frequently carried out for marketing purposes rather than genuine medical interest [2].

Moreover, PASs should be considered as post-authorization measures that are particularly useful in the context of special populations such as pediatric patients and those affected by rare diseases. The importance of PASs in the context of pediatric medicines lies in the fact that children are typically excluded from pre-authorization clinical trials due to ethical considerations and often respond to medicines differently from adults, highlighting the need for comprehensive investigations into the long-term effects, appropriate dosages, and potential side effects specific to children [5,6]. In addition, some of the PASs conducted in the pediatric population were imposed by regulators based on specific criteria such as the mechanism of action or the potential adverse events of the drug. Concerning rare diseases, where treatment options are limited and adverse effects might not have been detected during pre-marketing studies on small populations, PASs become a lifeline, offering an opportunity to gather real-world evidence and refine therapeutic approaches [7,8].

To strengthen the monitoring of the benefit–risk profile of medicines, the European Network of Centers for Pharmacoepidemiology and Pharmacovigilance (ENCePP) has been established and is coordinated by the European Medicines Agency (EMA). Its main objective is to facilitate the conduction of high-quality, multi-center, independent PASs with a focus on observational research.

The European Union electronic Post-Authorization Study Register (EU PAS Register) was a publicly available repository developed and supported by the EMA through the ENCePP containing more than 2000 PASs, and it has recently been replaced by the Head of Medicines Agencies (HMA)-EMA Catalogue of real-world data studies [9].

In 2019, the ENCePP Working Group 3 (WG3) “Data Sources and Multi-source Studies” carried out a detailed overview of the studies included in the EU PAS Register from its inception to 31 December 2018, with a focus on the multi-database studies [7].

To the best of our knowledge, no studies have identified and described the impact of PASs conducted in the pediatric population. Hence, the aim of this work was to analyze pediatric PASs registered in the ENCePP EU PAS Register to identify trends in terms of timing, age groups, and therapeutic areas and to discuss pediatric specificities, dealing with various aspects of the study design. The results of this analysis can be used to raise awareness of existing gaps in the post-authorization phase of pediatric medicines and to help industry and not-for-profit organizations find solutions to address unmet needs. By identifying areas in pediatric research that require further attention, this study seeks to guide stakeholders in effectively prioritizing resources and enhancing the quality and impact of pediatric post-marketing surveillance.

## 2. Materials and Methods

### 2.1. Data Collection

The data source for this study was a dataset provided by the EMA containing all PASs registered in the EU PAS register from September 2010 to 30 April 2023.

Data collection focused exclusively on PASs related to the pediatric population, excluding all studies involving both adult and pediatric patients. The sample size was determined using information about the age of the study population, thus filtering out PASs in the EMA dataset conducted in the pediatric population only (from preterm newborns to adolescents—17 years old).

Data collection started in October 2023 and was conducted independently by four researchers (A.L., A.D., A.D.E., G.S.G.). Each researcher collected data for a specific set of PASs. To standardize and harmonize the data collection procedure, a case report form and an instruction document were developed.

The publicly accessible EU PAS Register website was searched to retrieve information concerning the availability of study protocol, the summary of study results, and the presence of related publications. If a link to the publication(s) was not available on the EU PAS Register website, a specific search was carried out in PubMed. To collect relevant data, priority was given to the information included in the English version of the study protocol uploaded to the ENCePP EU PAS Register, where available. For studies included in a Risk Management Plan (RMP), PASs categories 1 (i.e., studies imposed as a condition to the Marketing Authorization) and 2 (i.e., studies imposed as a specific obligation in the context of a Marketing Authorization under exceptional circumstances) were used, and when the study protocol was not available, data were searched on the EMA website. When available, study reports and/or study synopses were used to retrieve relevant data. In the case study protocol, the study report, synopsis, and/or publication were missing; the information was collected by consulting the EU PAS Register webpage reporting information on each PAS. In case of conflicting information, priority was given to the study protocol.

Data collected for each study is detailed in Table S1. In brief, the information encompassed data concerning study identification (e.g., study title, brief description of the study, status of the study), study scope, countries involved, study type (e.g., observational studies, clinical trials), study design (e.g., descriptive study, cohort studies, cross-sectional studies), study objectives (e.g., disease epidemiology, risk assessment), funding sources, age range of the subjects enrolled, their classification in pediatric age groups (i.e., preterm newborn infants, term newborn infants, infants and toddlers, children and adolescent), and information on special study populations involved (e.g., lactating mothers). The concerned therapeutic area was assigned to each study based on the Anatomical Therapeutic Chemical (ATC) classification system. The number of drugs/classes under study was collected; however, it was agreed to limit the collection of information to the most relevant three drugs/drug classes included in this study. Data were gathered for each drug/class of drug, including the name, brand name, ATC code, type, and indications of whether it is a vaccine or an orphan drug. Additionally, the use of primary or secondary data sources for observational studies was reported. The detailed information collected for each study is listed in Table S1.

Moreover, an in-depth look at the different types of funding sources is provided in Table S2.

Once all the data were collected, a two-stage validation phase was carried out by cross-checking information retrieved for a 15% random sample of the identified studies. All cases of ambiguity and/or inconsistency were resolved through discussion, and if consensus was not reached, through the exploration of additional data sources (e.g., the literature search) or the involvement of a fifth expert (M.F.).

### 2.2. Statistical Analysis

A descriptive analysis was conducted on collected data concerning the identified PASs. Characteristics of study design, eligibility criteria, methodological aspects, study conditions and targets, and source of funding were evaluated and stratified by year of study registration. PASs were grouped by the year of registration. The percentage change in the summarized number of PASs from 2010 to 2023 was determined using the observed annual percentage values. Univariate Linear Regression analysis was used to evaluate trends in PAS registration over time. Linear regression beta coefficients were determined to evaluate potential changes in the number of PASs over time. This statistical analysis approach was also applied to the subsets of data regarding three age categories. To understand more about additional characteristics of the PASs in our data set, we also analyzed information on the studies’ source of funding and therapeutic area.

Unless specified otherwise, all statistical tests were two-sided and were performed using a significance (alpha) level of 0.05. Data were analyzed using the SPSS package (IBM SPSS Statistics for Windows, version 29.0, IBM Corp., Armonk, NY, USA).

## 3. Results

Out of the 2574 PASs extracted from the EU PAS Registry from its inception until 30 April 2023, 165 PASs were included in our analysis as they were conducted exclusively on the pediatric population. Out of 165 PASs, 45 were conducted exclusively in countries outside the EU.

At the time of the analysis, 95 (57.6%) PASs were finalized; 49 (29.7%) were ongoing, and 21 (12.7%) were planned (Table 1). Most studies (142; 86.1%) were observational, followed by systematic reviews/meta-analyses (7, 4.2%) and surveys (9, 5.5%). The scope was most frequently risk assessment (82, 49.7%), drug utilization (39, 23.6%), or effectiveness evaluation (35, 21.2%). It is important to note that each study could have more than one study objective.

Moreover, almost three-quarters of the PASs (120; 72.7%) were conducted in at least one European country. Half of the studies (83; 50.3%) were requested by regulatory authorities. Of all studies, 60 (36.4%) were part of a European RMP, while 10 studies (6.1%) were part of a non-EU risk management plan.

Of all 142 observational studies, half (76, 53.2%) used secondary data sources, while a third (48, 33.8%) used primary data collection.

Study results and publications could be gathered for 70% of the studies (116/165) and were considered primary sources of information together with the study protocol, when available.

An overview of all 165 pediatric PASs is also provided below using descriptive statistics.

The annual number of registered PASs increased significantly between 2010 and 2023, with a peak in 2014 (Table 2).

**Figure 1:**
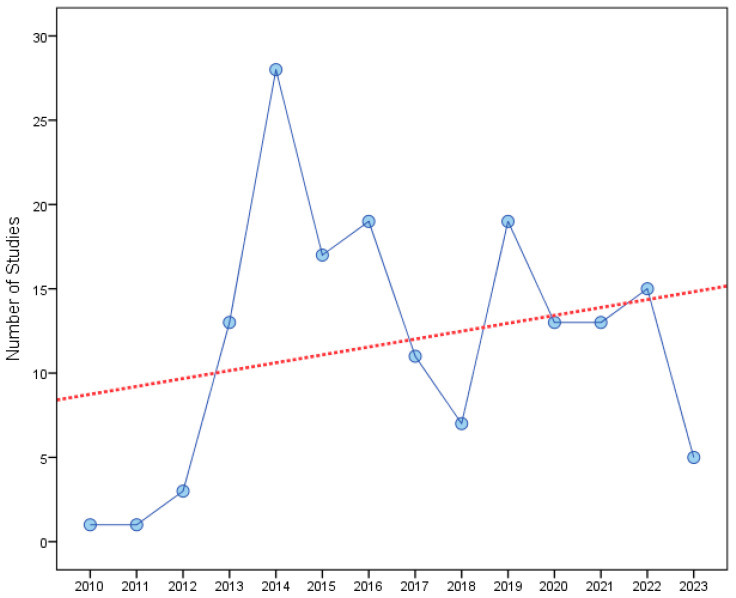
Number of registered PASs by year among all 165 included PASs. The red line represents the regression line estimated by the model.

In the linear regression on the relationship between the number of registered PASs (dependent variable) and the year of registration (independent variables X), the regression coefficient of 0.47 informed that the number of registered PASs increased by 0.47 with each additional year (Figure 1). A correlation coefficient (R) of 0.251 showed a positive linear relationship between the number of registered PASs and the year of registration; however, the relationship was non-significant (regression *p*-value = 0.386).

Most of the pediatric PASs (103, 62.4%) were funded by pharmaceutical companies, followed by those funded by government bodies (25, 4.1%) (Table 3).

When the study registration year was described by funding source, no clear time trends were identified by funding source, apart from the increase in PASs from 2011 to 2013, in PASs funded both by pharmaceutical companies and government agencies (Table 4).

With reference to the therapeutic area, anti-infective drugs for systemic uses (41, 24.8%), medicines for the nervous system (29, 17.6%), and antineoplastic and immunomodulating agents (24, 14.5%) were the most studied drugs (Table 5). In particular, among anti-infective drugs, 29 vaccines, seven antivirals, two antibacterials, two immune sera, immunoglobulin drugs, and one antimycotic were found. The medicines for the nervous system were instead mainly psychoanaleptics (12), followed by eight antiepileptics, six psycholeptics, two analgesics, and one anti-Parkinson drug.

Moreover, it is relevant to note that the number of study drugs under investigation varies widely among the PASs, and that sometimes PASs focus on classes of drugs (e.g., a class of vaccines or antibiotics) rather than on specific drug(s). Most, 67.9% (112/165), of the PASs focused on one study drug, 3% (5/165) on two, 4.8% (8/165) on more than three, while 13.3% (22/165) focused on an entire class of medicines. For the 10.9% (18/165), no specific study drug was considered since the focus of the PAS was on the disease.

With reference to the age groups, preterm newborns were involved in 43 studies (26%); term newborns (from 0 to 27 days) were involved in 71 (43%), infants and toddlers (from 28 days to 23 months) in 102 (61.8%), children (from 2 to 11 years) in 121 (73.3%), and adolescents (from 12 to 16/18 years, dependent on region) were included in 94 (57%) of the analyzed PASs.

**Figure 2:**
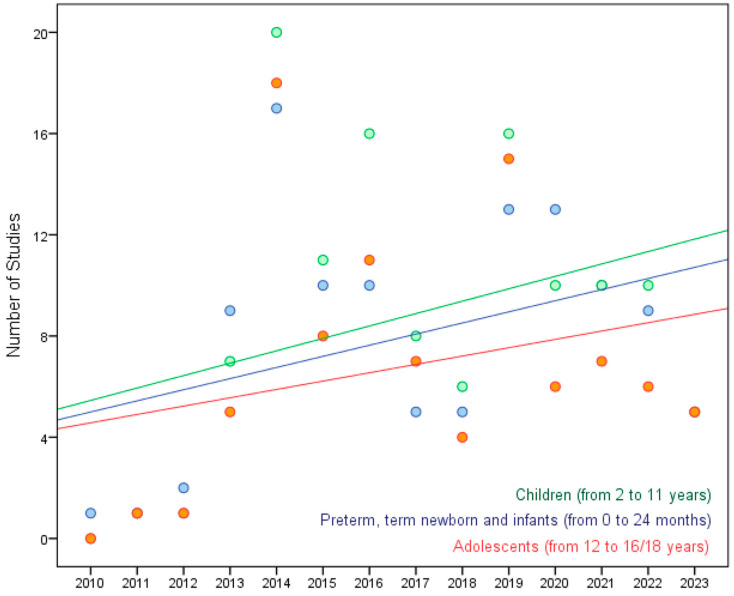
Number of registered PASs by year and age category among all 165 included PASs. The colored lines represent the regression lines of the three groups estimated by the model.

The results of the linear regression model, considering the age categories (i.e., preterm, term newborns and infants, children, adolescents), indicated an increasing trend in the number of registered PASs over time in all age groups (Figure 2; all the regression coefficients were positive: preterm/term newborn and infants 0.44 with R^2^ = 0.144; children with a regression coefficient 0.49 with R^2^ = 0.118; adolescents 0.33 with R^2^ = 0.073).

The greatest increase in the number of registered PASs over time (0.49/year) is observed in children; a slightly smaller increase (0.44/year) is observed in preterm/term newborns, while a smaller increase (0.33/year) is shown in the adolescent group followed by a decrease after 2019.

## 4. Discussion

An analysis of the pediatric PASs, as retrieved from the EU PAS register from its inception till April 2023, was performed using descriptive statistics to identify trends in terms of timing, age groups, and therapeutic areas and to discuss pediatric specificities. Sources of funding for the PASs were investigated as well.

During the collection phase, a systematic approach was followed to retrieve information from the main data sources and store it in a harmonized and standardized way to facilitate the analysis. Some challenges were encountered during this phase, mainly related to the unavailability of study protocols for some studies, leading to incomplete data retrieval or difficulties in interpreting the information available on the EU PAS Register website for those studies without the disposal of additional protocol information. These represent the main limitations of our work. Nevertheless, these challenges were overcome by consulting the available study documents (e.g., the summary of the study results) or by looking at the available publications related to the studies. Moreover, all cases of inconsistency in data collection were addressed through discussion, exploration of additional data sources, and reaching consensus among the involved researchers.

The results of our study provide valuable insights into the evolving landscape of pediatric pharmacovigilance and pharmacoepidemiology. In particular, the increase in the number of pediatric PASs registered over the years underlines the growing attention that the healthcare community, regulators, and pharmaceutical industry paid to the pediatric population and the efforts made to investigate and ensure the safety and effectiveness of medicines developed for this vulnerable population. The increase in the number of registered PASs reflects a concerted effort to address the regulatory and ethical imperatives surrounding the development and use of medicines in children, encouraged by the entry into force of the Pediatric Regulation in January 2007 [10]. The utilization of linear regression to explore the temporal trend in the identified PASs further elucidates this progression, revealing a statistically significant annual increase of 0.47 PASs per year. This finding not only highlights the proactive approach taken by regulatory authorities and pharmaceutical companies but also emphasizes the ongoing commitment to pediatric pharmacovigilance, thereby fostering safer medication practices for children. Moreover, the increase in the number of these pediatric studies, together with the proportion of observational studies, indicates that observational studies are increasingly considered to complement the available evidence from pediatric clinical trials. This represents an indicator of the direction in which clinical research is going, thus increasing the observational and post-marketing research to complement trial evidence.

Interestingly, out of 165 PASs, 45 were conducted exclusively in countries outside the EU. At first glance, this may seem confusing, as the EU PAS Register is an EU register; thus, only studies conducted in EU Member States could have been registered. However, it is important to clarify and emphasize that the EU PAS Register welcomed the registration of all pharmacovigilance and pharmacoepidemiology studies, regardless of the countries in which they were conducted. This inclusive approach underlines the Register’s commitment to providing a comprehensive overview of research efforts in these areas, ensuring that valuable data from a wide range of geographical locations are available to relevant stakeholders for analysis and decision-making. This is something that the EU will continue with the new HMA-EMA Catalogue, which is open for registration to all studies regardless of the country(ies) in which they are conducted [11]. Furthermore, it is important to emphasize that for the purposes of the analysis, EU Member States were classified according to the European situation in 2023, so the UK was not considered an EU country.

Of relevance is also the classification of the 165 pediatric PASs according to their source of funding (public or private), which provides valuable insights into the financial dynamics shaping pediatric pharmacovigilance efforts. As expected, the majority of PASs (62.4%) were funded by pharmaceutical companies, highlighting the key role of the industry in driving research in this area. Conversely, the comparatively lower proportion of PASs funded by not-for-profit organizations (e.g., universities) and public funds (28.5%) highlights a potential gap in public sector support for pediatric pharmacovigilance initiatives. The same trend is confirmed when looking at funding over time; while PASs funded by pharmaceutical companies show an increase, a similar trend is not observed for studies funded by other institutions. This discrepancy may be potentially driven by regulatory requirements reflecting different priorities and funding dynamics between the private and public sectors, and it underlines the need for joint efforts to ensure comprehensive and balanced pediatric drug safety research. Industry–academia collaborations should be encouraged, especially in areas of unmet therapeutic need to speed up scientific research. Regulatory guidance is deemed necessary to enhance collaboration in the pediatric field.

Moreover, while the EU PAS register is comprehensive in its coverage of study data on the source of funding, there is a notable lack of information on the study sponsorship, especially regarding whether the research is conducted by for-profit or not-for-profit organizations. This omission could pose a significant challenge in assessing the purpose of this study, as it becomes difficult to determine whether the intention behind the research is for-profit or not-for-profit. This shows that without transparency and a clear understanding of the aims and objectives of a study, it is difficult to make well-informed conclusions about PASs in pediatrics. To overcome this limitation, and considering the evolving landscape for the PASs currently included in the new HMA-EMA Catalogue of real-world data studies [9], we strongly recommend that this information is also mentioned for each study. In this way, stakeholders can gain a clearer understanding of the aims and objectives behind each study, thereby increasing transparency and facilitating informed decision-making within the scientific community.

With regard to the results concerning the distribution of PASs by therapeutic area, the majority of study drugs/classes of drugs under investigation belonged to the following three: anti-infectives, antineoplastic and immunomodulating agents, and drugs for the nervous system. This is partially in line with the main pediatric medicines studied in pre-marketing studies, namely, anti-infectives, antineoplastic, and immunomodulators. In fact, a systematic review by Deejesh Subramanian et al., 2022, found that while oncology drugs were widely represented in early-phase clinical trials, the most studied drugs in phase I-III pediatric trials were antivirals (27% of all trials), dominated by HIV/AIDS drugs, followed by blood products and modifiers (10%), drugs for genetic or enzyme protein disorders (8%), immunological agents (7%), and antibacterials (6%) [12]. The anti-infectives for systemic use representing the group with the highest ratio (21%) of the total number of pediatric authorized medicines are also confirmed by an analysis performed by Toma M and colleagues in 2021 [13].

This outcome suggests that it would be necessary to increase the percentage of PASs in certain therapeutic areas that are less represented, but it could be worth considering that this result may contain a source of bias, as some diseases do not affect the pediatric population, and, therefore, some therapeutic areas may be less represented.

Finally, looking at the pediatric age groups and discussing pediatric specificities, our analysis shows a difference in the number of PASs reported over time. Specifically, the number of studies reported for children (aged 2–11 years) shows an increasing trend over time, which is higher than the slightly increasing trend for adolescents (aged 12–16/18 years). This may give rise to different considerations, mainly related to the differences that exist among Member States with regard to the adolescent group. Firstly, the age of legal capacity varies among Member States, allowing, for example, for the inclusion of subjects over 16 years of age in adult clinical studies and, thus, excluding them from pediatric studies and, therefore, a less need for additional post-authorization data. Secondly, it may be easier to conduct PASs than pre-marketing studies in children, which raises more ethical and regulatory concerns and more recruitment difficulties. It may also be due to the fact that the adolescent population is often studied with the adult population, and our analysis, which focuses on studies conducted exclusively in the pediatric population, may not cover this sample. Moreover, as highlighted in the paper by Toma M and colleagues [13], most of the medicines were tested in the adolescent age group in the pre-marketing setting.

It is relevant to highlight that the regulatory landscape related to medicinal products is currently evolving. On 19 March 2024, the Committee on the Environment, Public Health and Food Safety (ENVI) at the European Parliament, in charge of the procedure, adopted its position on the new Directive and Regulation on medicinal products for human use and the proposed Directive also includes specific mention of pharmacovigilance to monitor long-term post-authorization safety and efficacy studies in children, including relevant data from off-label use of the product. The dossier will be taken up by the Parliament [14,15].

## 5. Conclusions

In conclusion, our results show that PASs, including observational studies, are increasing over time to complement the results of pre-marketing pediatric clinical trials. Moreover, greater availability of study documents for each study, including study protocols and results, is needed to have a better understanding of the level of detail of the information available on each study and to improve the transparency of pediatric studies. This could be implemented in the new HMA-EMA Catalogue of real-world data studies. Finally, collaborations between industry and academia should be promoted, and regulatory guidance is needed to prioritize research where there is a major unmet therapeutic need in pediatric research in terms of pediatric populations and therapeutic areas less studied in the post-authorization phase.

## Figures and Tables

**Table 1:** Characteristics of the 165 PASs on pediatric populations registered in the EU PAS Register from its inception to 30 April 2023.

	Studies on Pediatric Populations Registered in the EU PAS Register N = 165 (%)
**Study type**	
Observational study	142 (86.1)
Survey	9 (5.5)
Review or meta-analysis	7 (4.2)
Clinical trial	4 (2.4)
Other	3 (1.8)
**Study design**	
Descriptive study	18 (10.9)
Cohort studies	103 (62.4)
Cross-sectional studies	8 (4.8)
Case-control studies	7 (4.2)
Other	14 (8.5)
More than 1	13 (8.0)
Unknown	2 (1.2)
**Status of the Study**	
Finalized	95 (57.6)
Ongoing	49 (29.7)
Planned	21 (12.7)
**Funding Details**	
Private	109 (66.1)
Public	45 (27.3)
Mixed	9 (5.5)
Unknown	1 (0.6)
Scope of the study *	
Risk Assessment	82 (49.7)
Drug Utilization	39 (23.6)
Effectiveness Evaluation	35 (21.2)
Disease Epidemiology	27 (16.4)
Other Scope	22 (13.3)
**ENCePP Seal**	
Yes	7 (4.2)
No	158 (95.8)
**Requested by a regulator**	
Yes	83 (50.3)
No	80 (48.5)
Unknown	2 (1.2)
**Country**	
EU	120 (72.7)
Non-EU	45 (27.3)
**RMP status**	
EU RMP 1	12 (7.4)
EU RMP 2	4 (2.4)
EU RMP 3	44 (26.6)
Non-EU RMP only	10 (6.1)
Unknown	13 (7.8)
Not applicable	82 (49.7)
Data collection **	
Primary data	48 (33.8)
Secondary data	76 (53.2)
Mixed	18 (12.7)
**Use of reference drug for formal comparison**	
Yes	36 (21.8)
No	129 (78.2)
Subjects age range ***	
Preterm newborn infants	43 (26.1)
Term newborn infants (0 to 27 days)	71 (43.0)
Infants and toddlers (28 days to 23 months)	102 (61.8)
Children (2 to 11 years)	121 (73.3)
Adolescents 12 to 16/18 ^₸^	94 (57.0)

* Each study could have more than one scope category; hence, the scope categories were not mutually exclusive. ** The data collection variable was calculated only with reference to the 142 observational studies. *** Each study could have more than one category of age range. ^₸^ The adolescent upper age limit depends on the region.

**Table 2:** Number and percentage of registered PASs by year among all 165 included PASs.

Year of Study Registration	Frequency	Percent
2010	1	0.6
2011	1	0.6
2012	3	1.8
2013	13	7.9
2014	28	17.0
2015	17	10.3
2016	19	11.5
2017	11	6.7
2018	7	4.2
2019	19	11.5
2020	13	7.9
2021	13	7.9
2022	15	9.1
2023	5	3.0
**Total**	**165**	**100.0**

**Table 3:** Number and percentage of registered PASs by funding source among all 165 included PASs.

Funding Source	Frequency	Percent
Pharmaceutical companies	103	62.4
Government body	25	15.2
EU funding scheme	5	3.0
Research councils	1	0.6
Other *	16	9.7
More than one	14	8.5
Unknown	1	0.6
**Total**	**165**	**100.0**

* The ‘other’ category included mainly universities, hospitals, and national grants as sources of funding.

**Table 4:** Number and percentage of registered PASs by funding source and year among 126 PASs funded by pharmaceutical companies or government bodies.

Year of Study Registration	Pharmaceutical Companies		Government Body	
	Count	%	Count	%
2011	0	0.0	0	0.0
2012	0	0.0	0	0.0
2013	3	27.3	3	27.3
2014	24	85.7	2	7.1
2015	9	52.9	4	23.5
2016	12	66.7	3	16.7
2017	7	63.6	3	27.3
2018	4	66.7	0	0.0
2019	12	80.0	3	20.0
2020	9	81.8	1	9.1
2021	9	69.2	1	7.7
2022	9	64.3	3	21.4
2023	5	100.0	0	0.0
**Total**	**103**	**68.2**	**23**	**15.2**

**Table 5:** Number and percentage of registered PASs by therapeutic area among all 165 included PASs.

Therapeutic Area	Frequency	Percent
A: Alimentary tract and metabolism	6	3.6
B: Blood and blood-forming organs	8	4.8
C: Cardiovascular system	3	1.8
D: Dermatologicals	1	0.6
H: Systemic hormonal preparation, excluding sex hormones and insulin	9	5.5
J: Anti-infective for systemic uses	41	24.8
L: Antineoplastic and immunomodulating agents	24	14.5
M: Musculoskeletal system	2	1.2
N: Nervous system	29	17.6
P: Antiparasitic products, insecticides, and repellents	1	0.6
R: Respiratory system	9	5.5
S: Sensory organs	1	0.6
V: Various	6	3.6
Others *	25	15.2
**Total**	**165**	**100.0**

* The ‘others’ category mainly included the subcategories “more than one therapeutic area”, “unknown therapeutic area”, and “not applicable”.

## Data Availability

The raw data used were collected from the EU PAS register, which contains public data, although the link is currently no longer active as the EU PAS register has been replaced by the Head of Medicines Agencies (HMA)-EMA Catalogue of real-world data studies [9]. Available online: https://catalogues.ema.europa.eu/catalogue-rwd-studies accessed on 23 September 2024.

## Supplementary Materials

The following supporting information can be downloaded at https://www.mdpi.com/article/10.3390/pediatric17010024/s1, Table S1: Information collected for each study, Table S2: Information collected for funding source.
