# Supplementary material for: Descriptive Analysis of Pediatric Studies Included in the European Union Post-Authorization Study Register from 2010 to 2023
Source: Pediatr Rep. 2025 Feb 16;17(1):24. doi: 10.3390/pediatric17010024 (PMC11858588; doi:10.3390/pediatric17010024)
Supplement: Supplementary file 1 [file pediatrrep-17-00024-s001.zip › pediatrrep-3372227-Supplementary Materials.pdf]

Supplementary Materials

Article

Descriptive analysis of Pediatric Studies included in the European Union Post-Authorization Study Register from 2010 to 2023

Annalisa Landi* †[1,2], Giorgio Reggiardo†[1,3], Antonella Didio[2], Annunziata D'Ercole[2], Adriana Ceci[1,2,3], Grace Shalom Govere[3], Donato Bonifazi[1,3], Fedele Bonifazi[1,2], Salvatore Crisafulli[4], Gianluca Trifirò[4], Florentia Kaguelidou[5], Katja M Hakkarainen[6], Katarina Gvozdanović[7], Francesco Barone-Adesi[8], Andrealuna Ucciero[9], Mariagrazia Felisi[1,3]

[1] TEDDY European Network of Excellence for Pediatric Research;
[2] Fondazione per la Ricerca Farmacologica Gianni Benzi Onlus;
[3] CVBF – Clinical Validation from Biopharmaceutical Findings;
[4] Department of Diagnostics and Public Health, University of Verona, Verona, Italy;
[5] Paris Cité University, INSERM, URP 7323 'Pharmacologie et évaluation des thérapeutiques chez l'enfant et la femme enceinte', AP-HP, Hôpital Robert Debré, Centre d'Investigations Cliniques, CIC1426, 75019, Paris, France;
[6] Epidemiology and Real-World Evidence, Parexel International, Gothenburg, Sweden;
[7] Pharmacoepidemiology department, Teaching Institute of Public Health "Dr. Andrija Stampar", 10 000 Zagreb, Croatia;
[8] Department of Translational Medicine, University of Eastern Piedmont, Novara, Italy;
[9] Hospital Pharmacy, University Hospital Maggiore della Carità, Novara, Italy;

† These authors contributed equally to this work
* Correspondence: al@benzifoundation.org (A.L.); Tel.: +39 080 902 6797

Table S1. Information collected for each study

Main category	Item	
**Study identification**	EU PAS Register number	
	Study title	
	Year of study registration	
	Year of last update	
	Brief Description of the Study	
	Risk Management Plan	
	ENCePP Seal	
	Study requested by a regulator	
	**Study type**	Observational study
		Survey
		Review or meta-analysis
		Clinical trial
		Other

	Status of Study
**Research centres**	Countries in which this study is being conducted
	At least 1 EU country
**Sources of funding**	Funding
	Funding details
	Funding specification
	PI employed by study funder
**Population under study**	Subjects Age
	Preterm newborn infants
	Term newborn infants
	Infants and toddlers
	Children
	Adolescents
	Subjects Sex
	Specia population lactating mother
	Special population renal impairment
	Special population hepatic impairment
	Special population immunocompromised
	Special population Other
	Specific other special population
	Number of subjects enrolled
**Targets of the study**	Product lifecycle
	Medical condition under study
	Therapeutic area
	No of drugs under study
	Study medicine1 name
	Study medicine1 brand name
	Study medicine1 type
	Study medicine1 vaccine
	Study medicine1 ATC code
	Study medicine1 orphan in EU

	Study medicine2 name	
	Study medicine2 brand name	
	Study medicine2 type	
	Study medicine2 vaccine	
	Study medicine2 ATC code	
	Study medicine2 orphan in EU	
	Study medicine3 name	
	Study medicine3 brand name	
	Study medicine3 type	
	Study medicine3 vaccine	
	Study medicine3 ATC code	
	Study medicine3 orphan in EU	
**Data**	Source of data	
	Data collection	
	Secondary data	
	Multiple database study	
**Methodological aspects**	**Study design**	Descriptive study
		Cohort studies
		Cross sectional studies
		Case control studies
		other
		More than 1
		Unknown
	Use of reference drug for formal	
	**Scope**	Disease epidemiology
		Risk assessment Drug
		Drug utilisation study
		Effectiveness evaluation
		Other
**Documents**	Publications/Study results available	
	Summary of study results	

Table S2. Information collected for funding source

Funding Source
**Pharmaceutical companies**
**Government body**
**EU funding scheme**
**Research councils**
**Other***
**More than one**
**Unknown**

* The 'other' category included mainly universities, hospitals and national grants as sources of funding
